# Humoral immunity and transcriptome differences of COVID-19 inactivated vacciane and protein subunit vaccine as third booster dose in human

**DOI:** 10.3389/fimmu.2022.1027180

**Published:** 2022-10-21

**Authors:** Yuwei Zhang, Mingxiao Yao, Xingyu Guo, Shanshan Han, Shu Zhang, Jinzhong Zhang, Xiangkun Jiang, Jianxing Wang, Ming Fang, Shuang Wang, Bo Pang, Xiaolin Liu, Zengqiang Kou, Xiaolin Jiang

**Affiliations:** ^1^ Infectious Disease Prevention and Control Section, Shandong Provincial Center for Disease Control and Prevention, Jinan, Shandong, China; ^2^ School of Public Health and Health Management, Shandong First Medical University & Shandong Academy of Medical Sciences, Jinan, Shandong, China; ^3^ Institute of Immunization and Prevention, Liaocheng Center for Disease Control and Prevention, Liaocheng, Shandong, China; ^4^ Shandong Provincial Key Laboratory of Infectious Disease Control and Prevention, Shandong Provincial Center for Disease Control and Prevention, Jinan, China

**Keywords:** COVID-19, third booster vaccine, variants of concern, humoral immunity, transcriptome analysis

## Abstract

Under the background of the severe human health and world economic burden caused by COVID-19, the attenuation of vaccine protection efficacy, and the prevalence and immune escape of emerging variants of concern (VOCs), the third dose of booster immunization has been put on the agenda. Systems biology approaches can help us gain new perspectives on the characterization of immune responses and the identification of factors underlying vaccine-induced immune efficacy. We analyzed the antibody signature and transcriptional responses of participants vaccinated with COVID-19 inactivated vaccine and protein subunit vaccine as a third booster dose. The results from the antibody indicated that the third booster dose was effective, and that heterologous vaccination with the protein subunit vaccine as a booster dose induced stronger humoral immune responses than the homologous vaccination with inactivated vaccine, and might be more effective against VOCs. In transcriptomic analysis, protein subunit vaccine induced more differentially expressed genes that were significantly associated with many important innate immune pathways. Both the homologous and heterologous boosters could increase the effectiveness against COVID-19, and compared with the inactivated vaccine, the protein subunit vaccine, mediated a stronger humoral immune response and had a more significant correlation with the innate immune function module, which provided certain data support for the third booster immunization strategy.

## Introduction

Coronavirus disease 2019 (COVID-19) is an emerging respiratory infectious disease caused by severe acute respiratory syndrome coronavirus 2 (SARS-CoV-2), which has had a serious impact on world public health and economic development (1). The worldwide pandemic of COVID-19 has brought vaccine development to the forefront in unprecedented ways. More than 100 vaccines have been in the stage of clinical trials, and more than 20 vaccines have been approved for field application or emergency use (https://www.who.int/publications/m/item/draft-landscape-of-covid-19-candidate-vaccines).

Since the advent of the smallpox vaccine, vaccines have had a profound impact on human health, and their impact on infectious disease-related morbidity and mortality is irreplaceable (2). However, several long-term follow-up studies found that the antibody titer against SARS-CoV-2 diminished over time after vaccination (3–6). What’s more, SARS-CoV-2 is constantly evolving, with many variants emerging globally. The World Health Organization (WHO) has identified several variants of public health significance with greater transmissibility and immune evasion ability as variants of concern (VOCs) (7, 8). The prevalence of VOCs poses new challenges to the protective efficacy of COVID-19 vaccines based on9 wild-type virus strains (9).

In order to control COVID-19 more effectively, many regions have prioritized the implementation of the third booster immunization among high-risk groups who have completed the basic immunization. The COVID-19 inactivated vaccines BBIBP-CorV (10) and CoronaVac (11), and the protein subunit vaccine ZF2001 (12) showed good safety and immunogenicity in clinical trials. The above three COVID-19 vaccines have been widely promoted in Shandong Province, and have become the recommended vaccines for the third booster immunization. However, there are limited studies on the humoral immunity of the third dose booster vaccine in the real population, and the impact of different booster vaccination strategies on the vaccination effect is still unclear.

The development of transcriptomics has provided a very suitable avenue for exploring and dissecting the mechanisms involved in complex biological systems (13). Several studies have linked transcriptomic responses to immunological measures of vaccine responses (14–18), which provides us with new ideas to explore the underlying mechanisms of vaccine-induced immune efficacy and duration. Therefore, this study established population cohorts vaccinated with inactivated vaccine and protein subunit vaccine as the third booster vaccine, respectively. We collected serum and PBMC samples from participants at different time points before and after booster vaccination, and performed a systematic review of apparent antibody signatures and microscopic transcriptomics to provide data support for booster immunization. This study has important implications for developing rational booster vaccination strategies.

## Materials and methods

### Study population

In November 2021, we recruited healthy individuals to participate in this study in Liaocheng City. The inclusion criteria for participants included three requirements (1): Healthy people aged 18-79 years who had completed primary immunization with the inactivated COVID-19 vaccine for more than 6 months; (2) No clear history of COVID-19 infection; (3) Local residents who easy to follow-up. All participants were divided into two groups for a third booster, one of which continued to receive the inactivated vaccine BBIBP-CorV and the other received the protein subunit vaccine ZF2001. We collected venous blood samples from participants before booster vaccination, 7 days, 14 days, and 28 days after booster vaccination. All participants have signed written informed consent, and this study was approved by the Ethical Approval Committee of Shandong Center for Disease Control and Prevention.

### Serum and PMBCs isolation

Procoagulant venous blood was centrifuged at 2500 rpm/min for 10 minutes to separate serum and stored at -80°C until testing. Peripheral blood mononuclear cells (PBMCs) were isolated from anticoagulant venous blood by density gradient sedimentation, ensuring a cell number of at least 10^6^, frozen in cell preservation medium containing 10% DMSO and stored in liquid nitrogen.

### VeroE6 cell line culture and isolation of SARS-CoV-2 virus

The VeroE6 cell line was cultured in DMEM medium supplemented with 10% FBS and 1% penicillin-streptomycin at 37°C, and passaged every other day. 100μl throat swab samples of COVID-19 patients were added to the single-layer VeroE6 cell of T-25 culture flask, and the cytopathic effect (CPE) was observed every day. The virus supernatant with CPE was passaged for 3 times. When the CPE reached about 100%, the supernatant was collected after freezing and thawing three times, and stored at -80°C. The TCID_50_ of different isolates were tittered on VeroE6 cells, and sequence were confirmed by Nextera^®^ XT Library Prep Kit (Illumina, USA) on Miseq instrument. Manipulations involving SARS-CoV-2 strains must be performed in a certified Physical Protection Level 3 (PC3) laboratory.

### Serum neutralization assay

We measured serum neutralizing antibody titers at 4 time points in all participants against the SARS-CoV-2 virus by neutralization assay. Briefly, serum samples were inactivated at 56°C for 30 minutes, and 2-fold serial dilutions were prepared in DMEM with 1% FBS, with 2 replicates for each dilution. The 100TCID50 of wild-type, beta, delta, and omicron (BA.2.3) SARS-CoV-2 strains were mixed with equal amount of inactivated serum for 1 hour and transferred to 96-well plates with VeroE6 cells. After 7 days of culture, the CPE was observed and recorded, and the neutralizing antibody titers was calculated according to the pathological changes of the cells with different serum dilution.

### Quantitative SARS-CoV-2 IgG detection

Serum samples were diluted 200-fold and IgG antibodies were detected using an indirect ELISA kit (Vazyme, China) based on spike protein of SARS-CoV-2 as required by the instructions. Standard curves were made according to the six standard substances with known antibody concentration provided in the kit, and OD values of the sample to be tested were converted into antibody concentration. The antibody concentration was calculated after three repetitions.

### Transcriptome sequencing

The concentration and integrity of total RNA extracted from PBMCs by RNeasy Mini Kit (Qiagen, Germany) were checked using the Qubit RNA Assay Kit in Qubit 4.0 Fluorometer (Life Technologies, USA) and the RNA Nano 6000 Assay Kit of the Bioanalyzer 2100 System (Agilent Technologies, USA) respectively. An rRNA-depleted cDNA library was prepared from 100 ng of RNA using Stranded Total RNA Prep Ligation with Ribo-Zero Plus kit (Illumina, USA). The final library size of 300bp was denatured and sequenced on the Illumina NextSeq 2000 platform to generate 100 bp paired-end reads.

### Differentially expressed genes (DEGs) identification

The quality control, trimming, and mapping of the RNA-seq raw fasta data to the human reference genome hg38 were performed in the CLC Genomics Workbench, and gene expression was measured in transcripts per million (TPM). The iterative edgeR (19) and limma (20) packages are used to calculate normalization factors. DEGs were filtered out according to p-value < 0.1 and 2^logFC_cutoff criteria and visualized as volcanoes and heatmaps by the pheatmap package in R software.

### PPI network construction

The initial PPI networks of inactivated vaccine and protein subunit vaccine mediated up-regulated and down-regulated of DEGs were constructed using the STRING database (STRING v11.5; https://string-db.org/), and the nodes and edges information of the initial network was visualized and analyzed in Cytoscape software (21). To narrow the scope of the study, we extracted characteristic genes from all DEGs using the MCODE plugin in Cytoscape software.

### Characteristic gene expression verification

To further validate the transcriptomic analysis results, we transcribed 2 µg of RNA to cDNA using the Evo M-MLV RT Mix Kit with gDNA Clean for qPCR (Accurate Biotechnology). Quantitative normalization and analysis of the relative expression levels of mRNA for the characterized DEGs were carried out by reverse transcription quantitative polymerase chain reaction (RT−qPCR) using SYBR^®^ Green Premix Pro Taq HS qPCR Kit (Accurate Biotechnology). The relative expression levels of characteristic DEGs were calculated using the 2^-ΔΔCt^ method with the expression level of β-actin as a reference. We designed primer sequences for characteristic DEGs in the PrimerBank database (https://pga.mgh.harvard.edu/primerbank/), and the detailed primer information is shown in **Table S1**.

### Function enrichment analysis of characteristic DEGs

The prospective Gene Ontology (GO) terms of characteristic genes identified by the PPI network analysis were identified using clusterProfiler 4.0 (22). The bonferroni-adjusted P < 0.05 was used as the cut-off criterion. GO terms were summarize by removing redundant GO terms using Revigo (http://revigo.irb.hr/).

### Weighted gene co-expression network analysis (WGCNA)

In this study, we constructed gene co-expression networks by weighted correlation network analysis, and analyzed transcriptome profiles from 12 third-dose booster vaccinators, as well as their vaccination characteristics through WGCNA package (23) in R software. The optimal beta values were confirmed by a scale-free fit index and the highest mean connectivity by performing a gradient test. We choose power = 4 as the soft threshold to ensure a scale-free network. Subsequently, the topological overlap matrix (TOM) was calculated to measure network interconnectedness, and then hierarchical clustering was used to identify gene modules whose gene expression was highly correlated with the vaccination characteristics from the gene co-expression network, and each module containing at least 30 genes (minModuleSize=30). The genes in significant modules were analyzed using CluGO from Cytoscape software. The bonferroni-adjusted P < 0.05 was used as the cut-off criterion.

### Statistical analysis

The mean with SEM was used to describe antibody titers, and statistical significances were analyzed by two-sided paired t-tests with log-transformation and ordinary one-way ANOVA using GraphPad Prism 8.0. Neutralizing antibody titers were converted to log2 titers for the calculation of geometric mean titers (GMT).

## Results

### Characteristic of samples

**Figure 1:**
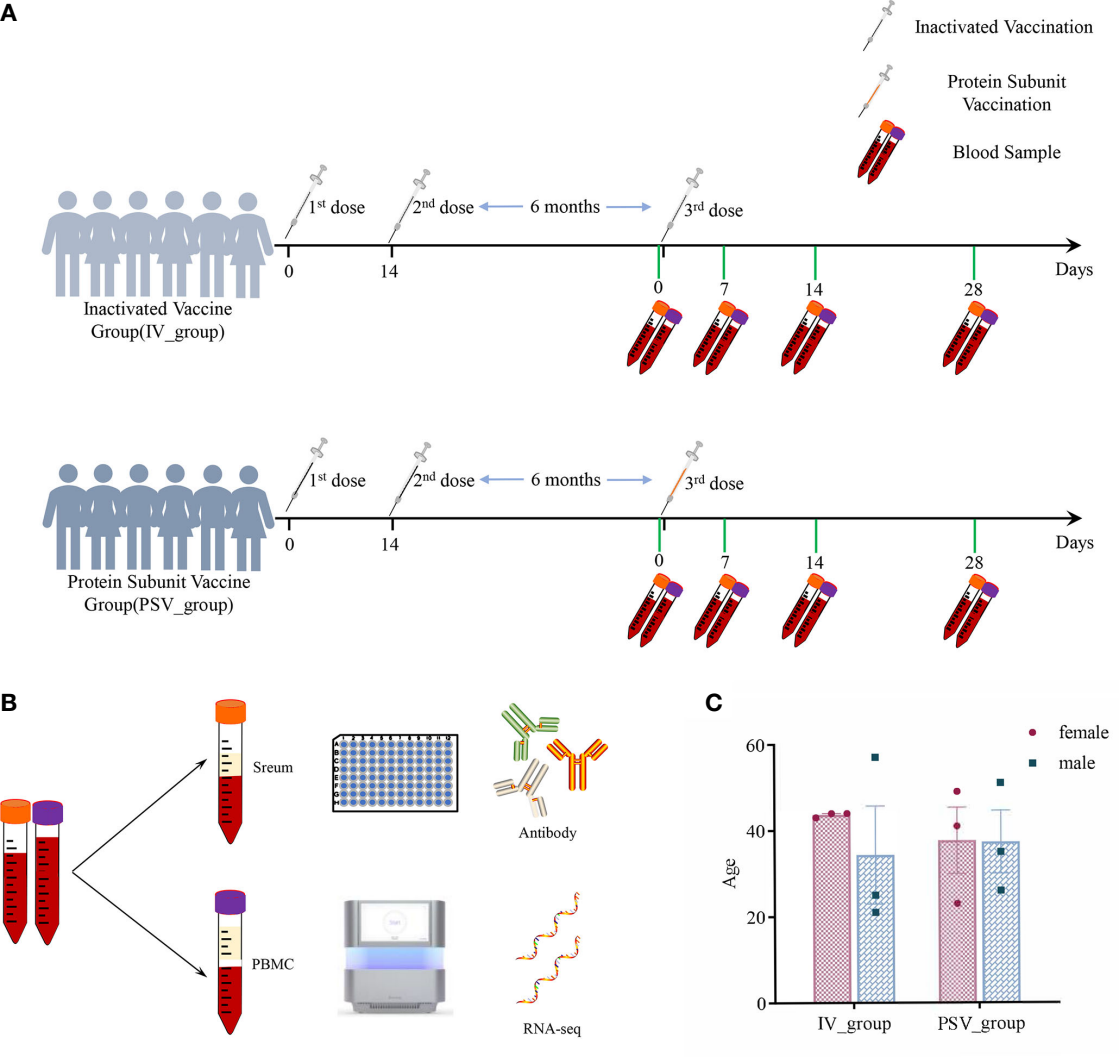
The details of this study design. **(A)** Participants’ vaccination and blood sample collection information. **(B)** Serum samples were used for antibody testing and PBMCs were used for transcriptome analysis. **(C)** Gender and age characteristics of participants.

Twelve healthy subjects without clear history of COVID-19 infection were enrolled in the study before receiving the third booster dose. The median age of all participants was 38 years (interquartile range IQR, 21–57), with a 50/50 gender mix. They are divided into two groups, including IV_group vaccinated with inactivated vaccine and PSV_group vaccinated with protein subunit vaccine. The mean booster interval of IV_group and PSV_group is 257 days (range: 244~269), and 262 days (range: 247~276), respectively, and there was no significant difference between the two groups for booster intervals (p=0.4438). We collected blood samples from participants before the third booster dose and at 7, 14 and 28 days after booster vaccination. Serum samples were used for neutralizing antibody detection, and PBMC samples were used for transcriptome system scanning and analysis. The specific details are shown in **Figure 1**.

### Characteristics of humoral immunity induced by the third booster vaccine

#### Serum sample neutralizing antibody titers

**Figure 2:**
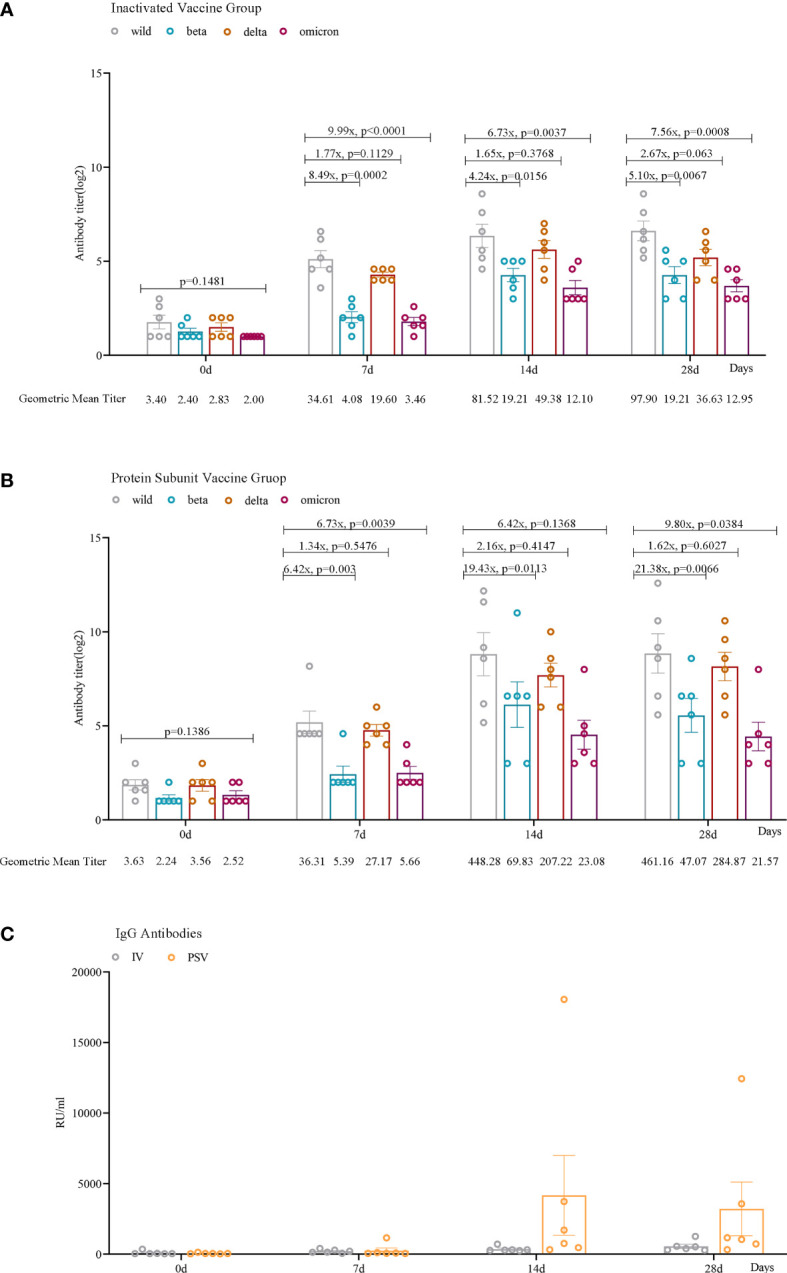
Humoral immune responses against wild, beta, delta, and omicron (BA.2.3) SARS-CoV-2 variants among participants. Neutralizing antibody levels against four lineages of SARS-CoV-2 in the IV_group **(A)** and PSV_group **(B)** before the third dose and 7 days, 14 days, and 28 days after vaccination. **(C)** IgG antibodies against SARS-CoV-2 in the IV_group and the PSV_group before the third dose and 7 days, 14 days, and 28 days after the vaccination. The geometric mean titer (GMT) and the fold difference in neutralizing antibodies between VOCs and wild-type are labeled. Geometric mean and SEM are depicted; statistical significances were analyzed by ordinary one-way ANOVA and two-sided paired t-tests with logtransformation.

Seven days after receiving the third dose of the inactivated vaccine, the GMTs of the participants’ sera against wild-type, beta, delta, and omicron (BA.2.3) variants were 34.61, 4.08, 19.60, and 3.46, respectively. After receiving the third dose of the protein subunit vaccine, the GMTs of serum against wild-type virus and beta, delta and omicron (BA.2.3) variants were 36.31, 5.39, 27.17 and 5.66, respectively. After 14 days of inoculation, the GMTs of the sera from two booster vaccination groups against the four SARS-CoV-2 variants increased to 81.52, 19.21, 49.38, 12.10 and 448.28, 69.83, 207.22, 23.08, respectively. After 28 days of the third booster dose, the GMTs of the sera from the two booster vaccination groups against the four SARS-CoV-2 variants were 97.90, 19.21, 36.63, 12.95 and 461.16, 47.07, 284.87, 21.57, respectively (**Figures 2A, B**).

#### The IgG titers of IV_group and PSV_group

We found that serum IgG antibody levels were lower before the booster vaccination. Antibody levels increased in all 12 participants 7 days after vaccination. Among them, 6 reached the maximum value after 14 days of inoculation, and attenuated slightly by 28 days, and the other 6 always maintained an upward trend. More importantly, the PSV_group had higher antibody levels at 14 days(mean=4170.39, SEM=2824.67) and 28 days(mean=3207.81, SEM=1903.07) than that in IV_group at 14 days(mean=355.05, SEM=71.31) and 28 days(mean=552.69, SEM=147.37) (**Figure 2C**).

### Transcriptional signature of PBMC induced by the third booster vaccine

#### Screening of DEGs

**Figure 3:**
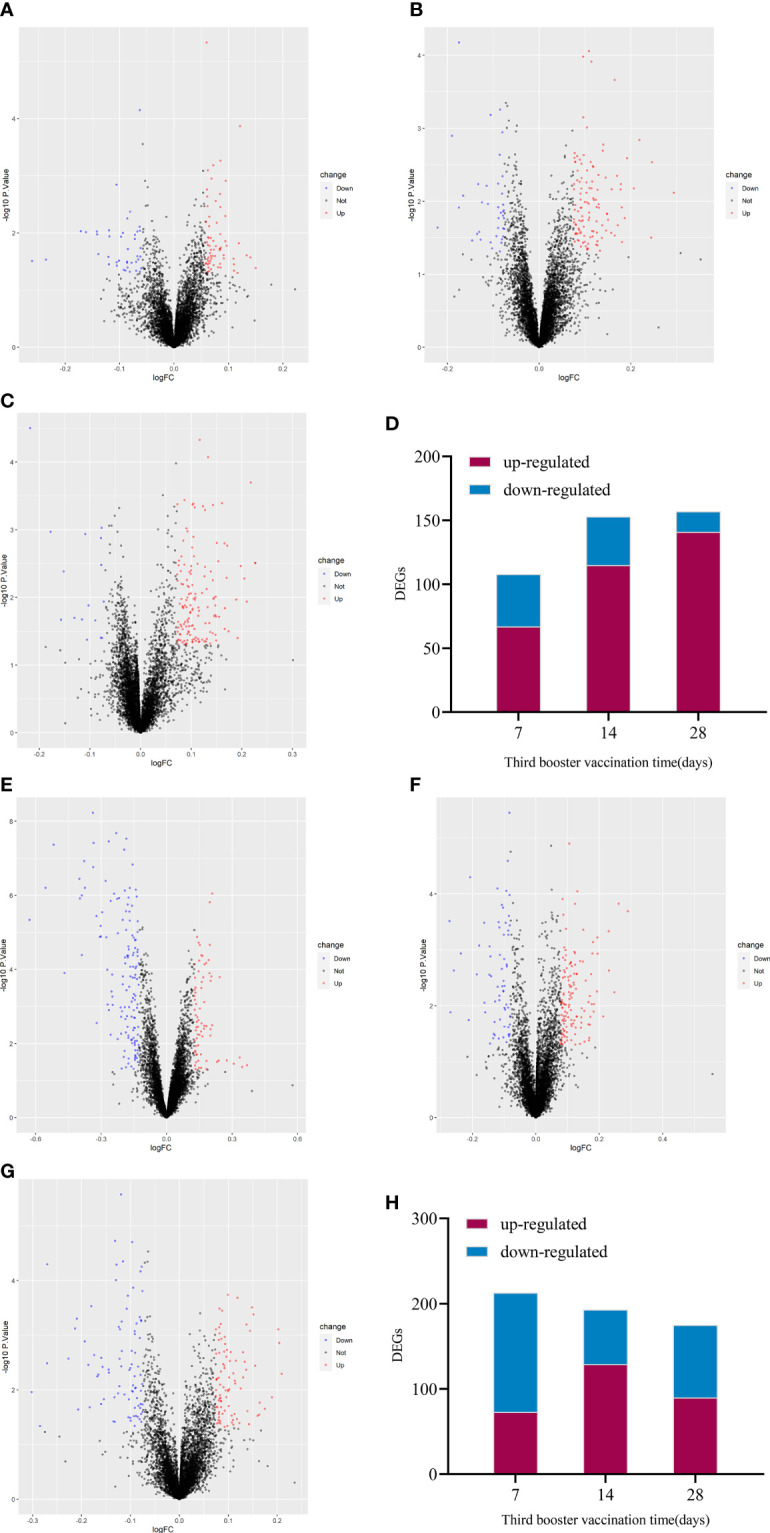
Volcano plot of global gene expression changes induced by the third dose of inactivated vaccine after 7 days **(A)**, 14 days **(B)** and 28 days **(C)** and the number of differentially expressed genes at three time points **(D)**. Volcano plot of global gene expression changes induced by the third dose of protein subunit vaccine after 7 days **(E)**, 14 days **(F)** and 28 days **(G)** and the number of differentially expressed genes at three time points **(H)**. Red dots indicate significant upregulation; blue dots indicate significant downregulation (p-value < 0.1 and 2^logFC_cutoff).

**Figure 4:**
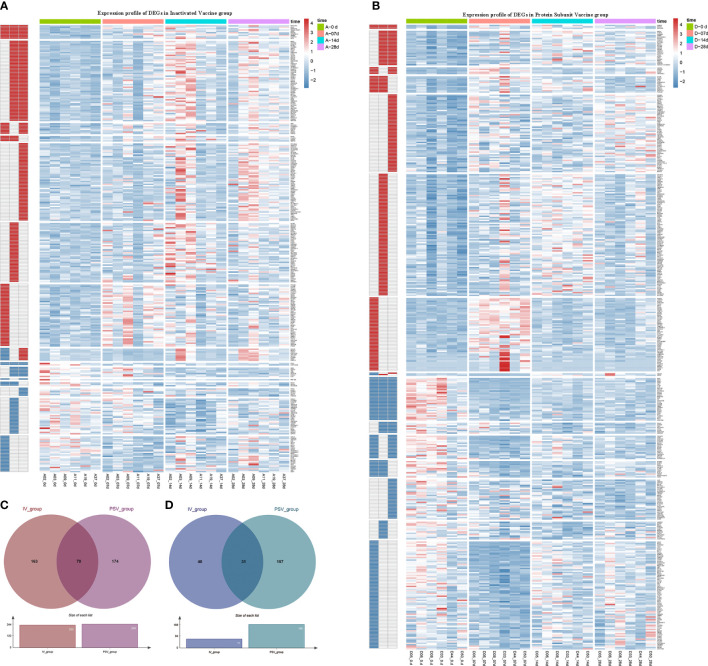
Hierarchical cluster analysis of DEGs in two vaccine groups. **(A)** The expression profile of 233 up-regulated DEGs and 71 down-regulated DEGs in IV_group. **(B)** The expression profile of 244 up-regulated DEGs and 188 down-regulated DEGs in PSV_group. Each row represents mRNA and each column represents a sample. Red indicates higher expression and blue indicates low expression in vaccination groups. Venn diagrams of up-regulated **(C)** and down-regulated **(D)** DEGs determined in IV_group and PSV_group.

The up-regulated DEGs of IV_group at 7d, 14d and 28d were 67, 115, 141, and the down-regulated DEGs were 41, 38, 16 respectively (**Figures 3A–D**). There were 73, 129, 90 up-regulated DEGs and 140, 64, 85 down-regulated DEGs in PSV_group after the third dose of vaccine 7d, 14d and 28d, respectively (**Figures 3E–H**). These results are shown in the volcano plot and histogram. Overall differential expression over time was shown in heatmap. In the IV_group, there were 233 up-regulated DEGs and 71 down-regulated DEGs in total (**Figure 4A**), while in the PSV_group, there were 244 up-regulated DEGs and 188 down-regulated DEGs in total (**Figure 4B**). The shared and unique up- regulated and down-regulated DEGs between the two groups were displayed with a venn diagram (**Figures 4C, D**). All DEGs were divided into 6 gene sets, including IV_group-specific, IV_group shared with PSV_group, and PSV_group-specific up-regulated and down-regulated gene sets, each gene set contained 163, 70, 174, 40, 31 and 157 DEGs, respectively.

### Prioritization of DEGs by PPI network analysis

**Figure 5:**
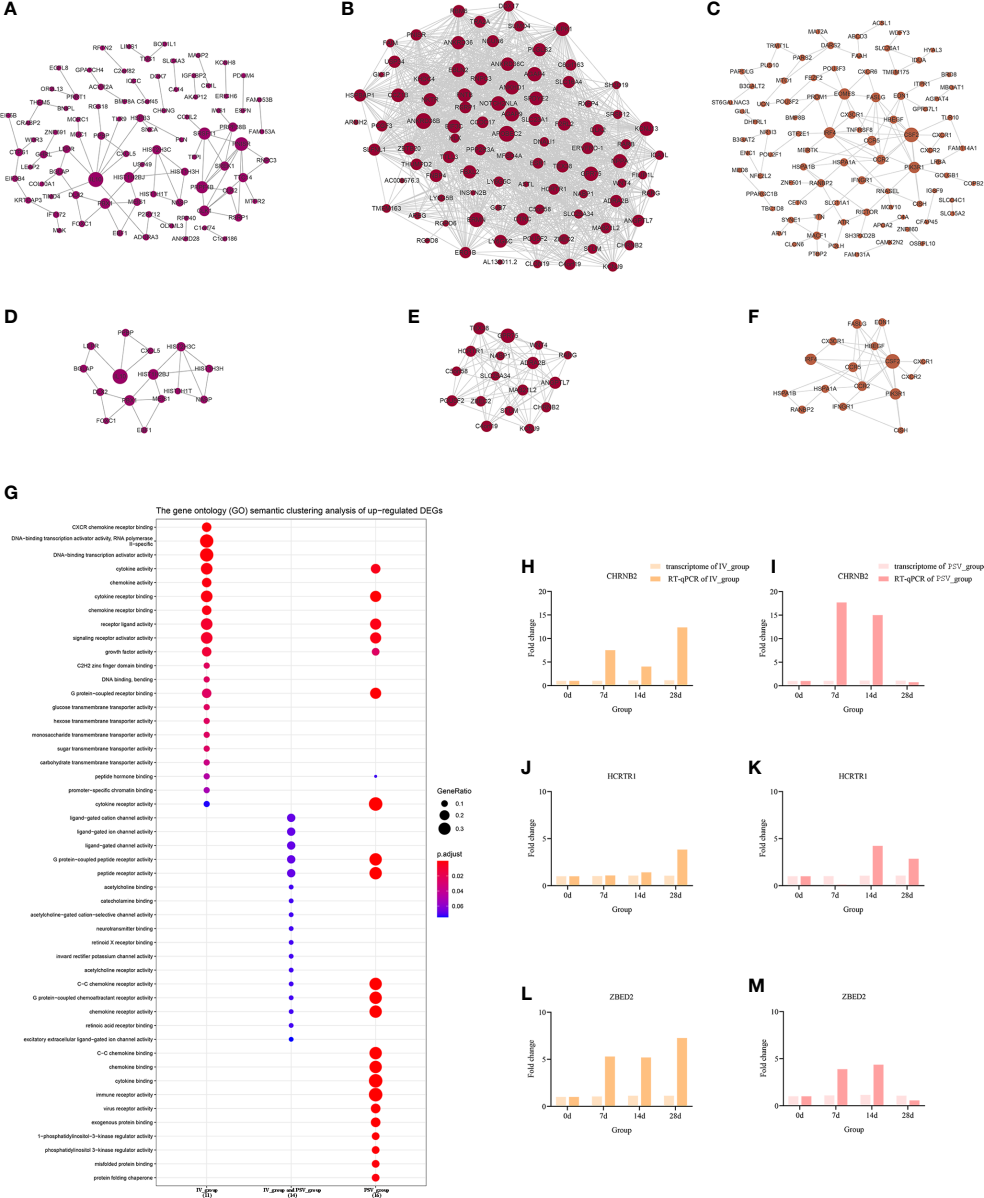
The up-regulated transcriptional changes induced by the third dose of inactivated vaccine and protein subunit vaccine. The PPI network of up-regulated gene sets in IV_group-specific **(A)**, IV_group shared with PSV_group **(B)** and PSV_group-specific **(C)** groups. The PPI network extracted from a-c by MCODE **(D–F)**. GO terms of the three core up-regulated gene sets extracted by MCODE **(G)**. The expression level verification of CHRNB2 **(H, I)**, HCRTR1 **(J, K)** and ZBED2 **(L, M)**.

**Figure 6:**
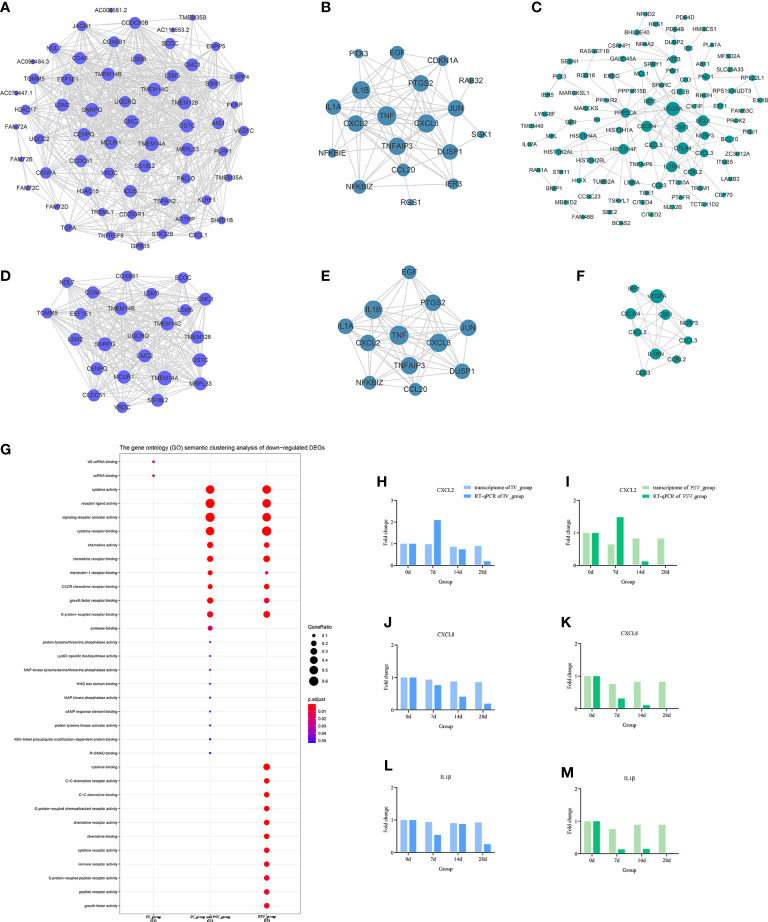
The down-regulated transcriptional changes induced by the third dose of inactivated vaccine and protein subunit vaccine. The PPI network of down-regulated gene sets in IV_group-specific **(A)**, IV_group shared with PSV_group **(B)** and PSV_group-specific **(C)** groups. The PPI network extracted from a-c by MCODE **(D–F)**. GO terms of the three core down-regulated gene sets extracted by MCODE **(G)**. The expression level verification of CXCL2 **(H, I)**, CXCL8 **(J, K)** and IL1β **(L, M)**.

To elucidate the interactions between the DEGs of each gene set, the node and edge relationships of the PPI network for each gene set were visualized in Cytoscape software (**Figures 5A–C**, **6A–C**). Using the k-core decomposition function of MCODE, 6 new sub-networks were created in which 15, 17, 16 up-regulated and 24, 12, 10 down-regulated DEGs were retained, respectively (**Figures 5D–F**, **6D–F**). The results of MCODE analysis of DEGs of the sub-networks are summarized in **Table S2** **-** **S7**.

### Verification of the relative expression levels of the characteristic DEGs

To further support the results of transcriptomic analysis, we screened six genes, CHRNB2, HCRTR1, ZBED2, CXCL2, CXCL8 and IL1β from the identified characteristic genes, and verified their relative expression by RT-qPCR at four time points before and after booster. The identification results showed that they were indeed differentially expressed in the vaccinated group, consistent with the bioinformatic analysis data (**Figures 5H–M**, **6H–M**), and most likely related to the host response mechanism to the vaccine, which requires further study.

### GO terms enrichment analyses of six sub-networks

The GO terms enrichment analyses were performed using clusterProfiler (**Figures 5G**, **6G**). Detailed enrichment information of GO terms after redundant removal with Revigo were summarized in **Tables S8**, **S9**. The up-regulated DEGs shared by IV_group and PSV_group were enriched in peptide receptor activity, ligand-gated ion channel activity and G protein-coupled peptide receptor activity, etc. The up-regulated DEGs specific to IV_group were enriched in DNA-binding transcription activator activity, RNA polymerase II-specific, chemokine receptor binding and signaling receptor activator activity, etc. The up-regulated DEGs mediated by PSV_group are enriched in chemokine binding, cytokine binding, immune receptor activity and virus receptor activity, etc. The down-regulated DEGs shared by IV_group and PSV_group were enriched in signaling receptor activator activity, interleukin-1 receptor binding, CXCR chemokine receptor binding and growth factor receptor binding and protease binding, etc. The DEGs of IV_group-specific down-regulated features were enriched in U6 snRNA binding and snRNA binding, etc. The PSV_group-specific down-regulated DEGs were enriched in cytokine binding, chemokine binding, CXCR chemokine receptor binding, growth factor receptor binding and cytokine receptor activity.

### WGCNA: Identify important feature modules associated with vaccination signatures

**Figure 7:**
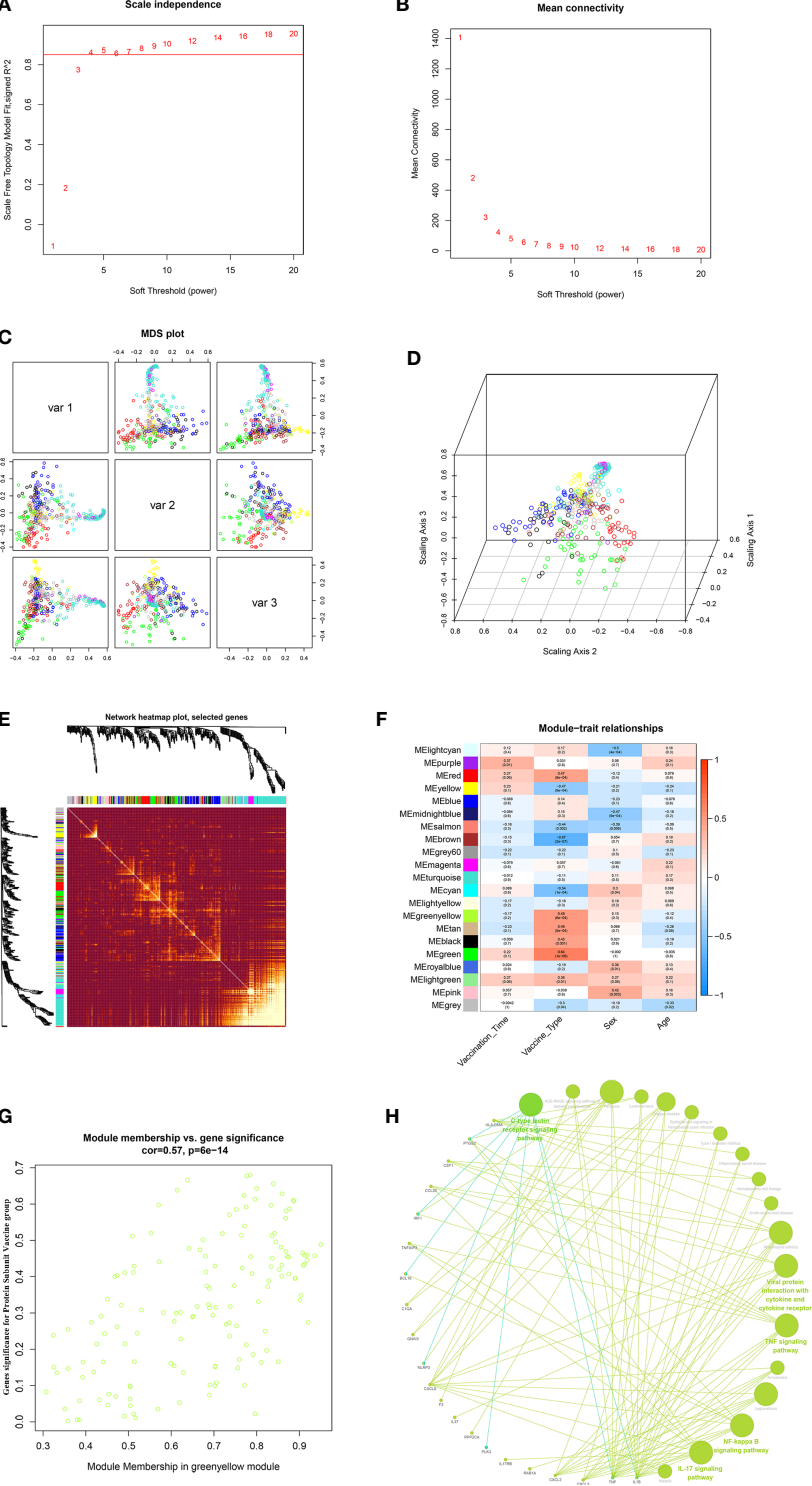
WGCNA of the PBMCs transcriptome. **(A, B)** Analysis of network topology for various soft-thresholding powers. The left panel shows the scale-free fit index (y-axis) as a function of the soft-thresholding power (x-axis). The right panel displays the mean connectivity (degree, y-axis) as a function of the soft-thresholding power (x-axis). **(C, D)** 3D cluster map of genes, based on differences in topological overlap, and assigned module colors. **(E)** Heatmap depicts the Topological Overlap Matrix (TOM) of genes selected for weighted co-expression network analysis. Light color represents lower overlap and red represents higher overlap. **(F)** Module-trait associations: Each row corresponds to a module eigengene and each column to a trait. Each cell contains the corresponding correlation and p-value. **(G)** Scatter diagram for MM vs GS in the greenyellow (eigengene value = 0.48). **(H)** KEGG signaling pathway enriched by greenyellow gene module.

In this study, to ensure the construction of a scale-free network, β was determined as a power of 4 (scale-free R^2^ = 0.85) as a soft threshold parameter (**Figures 7A, B**). In the transcriptome data, a total of 21 modules were identified by hierarchical clustering and minModuleSize=30, and the dendrogram was clustered based on the dissimilarity measure (1-TOM) (**Figures 7C–E**). The correlation between the vaccination signatures and the co-expression module is shown in **Figure 7F**, where the greenyellow (eigengene value = 0.48) module is significantly positively correlated with the booster type (**Figure 7G**). The functions of the hub genes in greenyellow module mainly focus on many immune-related KEGG signaling pathways (**Figure 7H**), and the detailed enrichment information of these pathways were shown in **Table S10**.

## Discussion

Traditional apparent antibody signatures can help us to directly evaluate vaccine effectiveness, and systematic scanning and analysis of vaccinated transcriptome data can elucidate the response mechanism of the host’s molecular immune response to vaccines (24). From the perspective of apparent antibody characteristics and microtranscriptomics, we systematically compared the differences in immune responses induced by the third booster immunization of inactivated vaccine and protein subunit vaccine after basic immunization with inactivated vaccine.

This study revealed the characteristics of apparent antibodies elicited by inactivated vaccine and protein subunit vaccine from multiple perspectives. From the time course of vaccination, with the prolongation of vaccination time, the level of neutralizing antibody gradually increased from 0 to 14 days, and the antibody level of individual participants decreased slightly at 28 days. The neutralization antibody titer of serum in the PSV_group was significantly higher than that in the IV_group. The dynamic changes of participants’ serum IgG antibodies exhibited similar characteristics with neutralizing antibodies. Under the current pandemic situation dominated by omicron variants, it is critical to analyze the neutralizing ability of serum after vaccination against different circulating SARS-CoV-2 variants. Consistent with the results of some previously published studies (25–27), the neutralization antibody titer of serum after booster vaccination against tested VOCs (beta and omicron) was significantly lower than that for the wild-type SARS-CoV-2. The results suggested that both the homologous and heterologous boosters could increase humoral immune responses against SARS-CoV-2, and heterologous booster with protein subunit vaccine induces a stronger humoral immune response than the homologous vaccination with inactivated vaccine and may be more efficient against VOCs.

Transcriptomic studies of PBMCs can provide a comprehensive summary of immune responses and have become important in immunology and infectious disease research tool (13, 28). Transcriptome analysis of PBMCs of the third booster dose showed that more DEGs were induced by protein subunit vaccine compared with the inactivated vaccine. The DEGs that they jointly up-regulated are involved in functional pathways such as macromolecular transmembrane and transport, which may be related to the synthesis and release of antibodies. On this basis, protein subunit vaccine activated more immune-related functions, such as cytokine binding; immune receptor activity; viral receptor activity and foreign protein binding, etc. Both vaccine-mediated down-regulated DEGs were enriched for signaling receptor activator activity, interleukin-1 receptor binding, CXCR chemokine receptor binding functional pathways, and protein subunit vaccine mobilized more suppression of innate immunity than inactivated vaccine. The inhibition of these immune functions may be related to the host’s feedback regulation and self-protection function to avoid excessive inflammation, and protein subunit vaccine activates more immune pathways and correspondingly more inhibition.

In order to more directly demonstrate the effect of the third dose of vaccine type on the transcriptome and reveal the different characteristics of PSV_group and IV_group transcriptome, we further carried out WGCNA on the transcriptomic data. The results showed that gene modules significantly related to PSV_group were mainly enriched in IL-17 signaling pathway, TNF signaling pathway, Rheumatoid arthritis, NF-kappa B signaling pathway, Pertussis, Legionellosis, Viral protein interaction with cytokine and cytokine receptor and other important innate immune functions. The innate immune function serves as the early defense line of the immune system, which can rapidly generate a stable response to antigens and adjuvants. In previous studies on transcriptional regulation of other vaccines, significant innate immune responses were also observed (14, 29, 30), and the activation of innate immune responses may be an important basis for the protective effects of vaccines.

Systems biology approaches can help us permit the observation of a global picture of vaccine-induced immune responses. Immunological antibody indicators and transcriptome system scan results together suggest that PSV_group mediates stronger humoral immune responses than IV_group and has a more significant correlation with innate immune function modules. Given the current results, protein subunit vaccine may be a better choice as a third booster vaccine, and a heterologous vaccination strategy may be better than continuing with the same type of vaccine. However, the evaluation of vaccine effectiveness and the formulation of reasonable vaccination strategies need to be more cautious, and longer-term observations in larger sample sizes are required.

## Data availability statement

The datasets presented in this study can be found in online repositories. The names of the repository/repositories and accession number(s) can be found below: https://www.ncbi.nlm.nih.gov/geo/, GSE206023; https://gisaid.org/, EPI_ISL_13499639~EPI_ISL_13499642.

## Ethics statement

All participants have signed written informed consent, and this study was approved by the Ethical Approval Committee of Shandong Center for Disease Control and Prevention (Ethical approval number: SDJK2022-003-01). The patients/participants provided their written informed consent to participate in this study.

## Author contributions

XLJ and YWZ conceived the study, developed the methodology, analyzed the data and prepared the manuscript. ZQK and XLJ secured funding. JZZ and XKJ recruited participants and collected samples. YWZ performed transcriptomics analysis and bioinformatics. YWZ, MXY, XYG, SSH, SZ, JXW, MF, SW, BP and XLL participated in neutralizing antibody experiments.

## Funding

This work was supported by the Major Scientific and Technological Innovation Project in Shandong Province (grant numbers: 2020SFXGFY02-1), Key Research and Development plan of Shandong Province (grant numbers: 2021RZA01021) and Natural Science Foundation of Shandong Province (grant numbers: ZR202112040005).

## Acknowledgments

This article was submitted as a preprint to The Lancet on SSRN (http://ssrn.com/abstract=4175229).

## Conflict of interest

The authors declare that the research was conducted in the absence of any commercial or financial relationships that could be construed as a potential conflict of interest.

## Publisher’s note

All claims expressed in this article are solely those of the authors and do not necessarily represent those of their affiliated organizations, or those of the publisher, the editors and the reviewers. Any product that may be evaluated in this article, or claim that may be made by its manufacturer, is not guaranteed or endorsed by the publisher.
