# Supplementary material for: Humoral immunity and transcriptome differences of COVID-19 inactivated vacciane and protein subunit vaccine as third booster dose in human
Source: Front Immunol. 2022 Oct 21;13:1027180. doi: 10.3389/fimmu.2022.1027180 (PMC9634958; doi:10.3389/fimmu.2022.1027180)
Supplement: Supplementary file 1 [file Table_1.doc]

**Table S1. Sequences of primers used.**

| Gene Symbol | primer-Forward (5'-3') | primer-Reverse (5'-3') |
| --- | --- | --- |
| CHRNB2 | CACCTGGAAGCCTGAAGAGTT | ATCATAGGAGACCACGGCATTG |
| HCRTR1 | CATCAGCGTCCTCAATGTCCT | GAAGGTGAAGCAGGCGTAGAC |
| ZBED2 | TAGTGAGACAGGAGAACTGGTTGG | TGGCATACTGGTTGGGATGGT |
| CXCL2 | ACAGTGTGTGGTCAACATTTCTC | TCTGCTCTAACACAGAGGGAAAC |
| CXCL8 | CACTGTGTGTAAACATGACTTCCAA | TGTGGTCCACTCTCAATCACTCTC |
| IL1β | CCAGGGACAGGATATGGAGCA | TTCAACACGCAGGACAGGTACAG |
| β-actin | CCTGGCACCCAGCACAAT | GCCGATCCACACGGAGTACT |
