# Supplementary material for: Humoral immunity and transcriptome differences of COVID-19 inactivated vacciane and protein subunit vaccine as third booster dose in human
Source: Front Immunol. 2022 Oct 21;13:1027180. doi: 10.3389/fimmu.2022.1027180 (PMC9634958; doi:10.3389/fimmu.2022.1027180)
Supplement: Supplementary file 2 [file Table_2.doc]

Table S2. The sub-network of IV_group-specific up-regulated genes.

| **Symbol** | **Degree unDir** | **MCODE::Clusters (1)** | **MCODE::Score (1)** |
| --- | --- | --- | --- |
| BGLAP | 3 | Cluster 0 | 0.50 |
| CXCL5 | 2 | Cluster 0 | 2.00 |
| DLX2 | 3 | Cluster 0 | 2.00 |
| EBF1 | 2 | Cluster 0 | 2.00 |
| FOXC1 | 2 | Cluster 0 | 2.00 |
| HIST1H1T | 2 | Cluster 0 | 2.00 |
| HIST1H2BJ | 5 | Cluster 0 | 1.20 |
| HIST1H3H | 3 | Cluster 0 | 1.67 |
| HIST2H3C | 4 | Cluster 0 | 1.40 |
| IL10 | 8 | Cluster 0 | 2.00 |
| LEPR | 3 | Cluster 0 | 0.50 |
| MEIS1 | 3 | Cluster 0 | 1.67 |
| NASP | 3 | Cluster 0 | 1.67 |
| PBX1 | 5 | Cluster 0 | 1.07 |
| PPBP | 3 | Cluster 0 | 2.00 |
