# Supplementary material for: Humoral immunity and transcriptome differences of COVID-19 inactivated vacciane and protein subunit vaccine as third booster dose in human
Source: Front Immunol. 2022 Oct 21;13:1027180. doi: 10.3389/fimmu.2022.1027180 (PMC9634958; doi:10.3389/fimmu.2022.1027180)
Supplement: Supplementary file 3 [file Table_3.doc]

Table S3. The sub-network of IV_group and PSV_group common up-regulated genes.

| **Symbol** | **Degree unDir** | **MCODE::Clusters (1)** | **MCODE::Score (1)** |
| --- | --- | --- | --- |
| ADRA2B | 39 | Cluster 0 | 7.78 |
| ANGPTL7 | 38 | Cluster 0 | 7.89 |
| C4orf19 | 34 | Cluster 0 | 8.12 |
| C5orf58 | 24 | Cluster 0 | 9.33 |
| CHRNB2 | 27 | Cluster 0 | 9.30 |
| GPR75 | 51 | Cluster 0 | 9.50 |
| HCRTR1 | 32 | Cluster 0 | 8.12 |
| KCNJ9 | 25 | Cluster 0 | 9.04 |
| MAB21L2 | 32 | Cluster 0 | 9.14 |
| NABP1 | 20 | Cluster 0 | 8.57 |
| POU5F2 | 33 | Cluster 0 | 9.68 |
| RARG | 17 | Cluster 0 | 8.79 |
| SLC25A34 | 22 | Cluster 0 | 9.61 |
| STUM | 25 | Cluster 0 | 8.34 |
| TEX38 | 39 | Cluster 0 | 9.68 |
| WNT4 | 21 | Cluster 0 | 9.30 |
| ZBED2 | 29 | Cluster 0 | 8.57 |
