# Supplementary material for: Humoral immunity and transcriptome differences of COVID-19 inactivated vacciane and protein subunit vaccine as third booster dose in human
Source: Front Immunol. 2022 Oct 21;13:1027180. doi: 10.3389/fimmu.2022.1027180 (PMC9634958; doi:10.3389/fimmu.2022.1027180)
Supplement: Supplementary file 4 [file Table_4.doc]

Table S4. The sub-network of PSV_group-specific up-regulated genes.

| **Symbol** | **Degree unDir** | **MCODE::Clusters (1)** | **MCODE::Score (1)** |
| --- | --- | --- | --- |
| CCR2 | 7 | Cluster 0 | 3.00 |
| CCR5 | 7 | Cluster 0 | 3.00 |
| CISH | 3 | Cluster 0 | 3.00 |
| CSF2 | 13 | Cluster 0 | 1.36 |
| CX3CR1 | 5 | Cluster 0 | 1.40 |
| CXCR1 | 2 | Cluster 0 | 2.00 |
| CXCR2 | 2 | Cluster 0 | 2.00 |
| EDN1 | 5 | Cluster 0 | 1.67 |
| FASLG | 7 | Cluster 0 | 3.00 |
| HBEGF | 5 | Cluster 0 | 1.20 |
| HSPA1A | 5 | Cluster 0 | 1.40 |
| HSPA1B | 4 | Cluster 0 | 1.67 |
| IFNGR1 | 5 | Cluster 0 | 3.00 |
| IRF4 | 9 | Cluster 0 | 1.05 |
| PIK3R1 | 9 | Cluster 0 | 3.00 |
| RANBP2 | 5 | Cluster 0 | 2.00 |
