# Supplementary material for: Humoral immunity and transcriptome differences of COVID-19 inactivated vacciane and protein subunit vaccine as third booster dose in human
Source: Front Immunol. 2022 Oct 21;13:1027180. doi: 10.3389/fimmu.2022.1027180 (PMC9634958; doi:10.3389/fimmu.2022.1027180)
Supplement: Supplementary file 5 [file Table_5.doc]

Table S5. The sub-network of IV_group-specific down-regulated genes.

| **Symbol** | **Degree unDir** | **MCODE::Clusters (1)** | **MCODE::Score (1)** |
| --- | --- | --- | --- |
| CCDC51 | 32 | Cluster 0 | 16.46 |
| CENPQ | 35 | Cluster 0 | 15.66 |
| CMC1 | 35 | Cluster 0 | 16.32 |
| CMC2 | 36 | Cluster 0 | 16.38 |
| COA6 | 33 | Cluster 0 | 17.19 |
| COX6B1 | 27 | Cluster 0 | 14.82 |
| EEF1E1 | 30 | Cluster 0 | 16.70 |
| LSM2 | 35 | Cluster 0 | 15.40 |
| LSM3 | 28 | Cluster 0 | 15.59 |
| LSM5 | 30 | Cluster 0 | 16.04 |
| MCUR1 | 37 | Cluster 0 | 16.32 |
| MRPL33 | 36 | Cluster 0 | 16.44 |
| NOL7 | 26 | Cluster 0 | 15.97 |
| OSTC | 38 | Cluster 0 | 17.19 |
| SCOC | 27 | Cluster 0 | 14.42 |
| SNRPG | 40 | Cluster 0 | 17.19 |
| SS18L2 | 35 | Cluster 0 | 17.19 |
| TMEM128 | 37 | Cluster 0 | 14.80 |
| TMEM14A | 42 | Cluster 0 | 16.86 |
| TMEM14B | 36 | Cluster 0 | 17.19 |
| TMEM14C | 38 | Cluster 0 | 17.42 |
| TOMM5 | 26 | Cluster 0 | 16.57 |
| UQCRQ | 29 | Cluster 0 | 16.57 |
| YRDC | 28 | Cluster 0 | 14.93 |
