# Supplementary material for: Humoral immunity and transcriptome differences of COVID-19 inactivated vacciane and protein subunit vaccine as third booster dose in human
Source: Front Immunol. 2022 Oct 21;13:1027180. doi: 10.3389/fimmu.2022.1027180 (PMC9634958; doi:10.3389/fimmu.2022.1027180)
Supplement: Supplementary file 6 [file Table_6.doc]

Table S6. The sub-network of IV_group and PSV_group common down-regulated genes.

| **Symbol** | **Degree unDir** | **MCODE::Clusters (1)** | **MCODE::Score (1)** |
| --- | --- | --- | --- |
| CCL20 | 8 | Cluster 0 | 6.81 |
| CXCL2 | 12 | Cluster 0 | 7.03 |
| CXCL8 | 14 | Cluster 0 | 7.03 |
| DUSP1 | 11 | Cluster 0 | 6.81 |
| EGF | 9 | Cluster 0 | 6.81 |
| IL1A | 11 | Cluster 0 | 6.24 |
| IL1B | 14 | Cluster 0 | 7.03 |
| JUN | 12 | Cluster 0 | 7.64 |
| NFKBIZ | 9 | Cluster 0 | 7.00 |
| PTGS2 | 12 | Cluster 0 | 7.64 |
| TNF | 15 | Cluster 0 | 7.03 |
| TNFAIP3 | 13 | Cluster 0 | 7.27 |
