# Supplementary material for: Humoral immunity and transcriptome differences of COVID-19 inactivated vacciane and protein subunit vaccine as third booster dose in human
Source: Front Immunol. 2022 Oct 21;13:1027180. doi: 10.3389/fimmu.2022.1027180 (PMC9634958; doi:10.3389/fimmu.2022.1027180)
Supplement: Supplementary file 7 [file Table_7.doc]

Table S7. The sub-network of PSV_group-specific down-regulated genes.

| **Symbol** | **Degree unDir** | **MCODE::Clusters (1)** | **MCODE::Score (1)** |
| --- | --- | --- | --- |
| CCRL2 | 6 | Cluster 0 | 3.00 |
| CD83 | 5 | Cluster 0 | 3.00 |
| CSF1 | 9 | Cluster 0 | 2.67 |
| CXCL3 | 6 | Cluster 0 | 3.73 |
| CXCL5 | 6 | Cluster 0 | 3.73 |
| CXCR4 | 10 | Cluster 0 | 4.00 |
| IL1RN | 10 | Cluster 0 | 4.00 |
| IRF1 | 6 | Cluster 0 | 3.00 |
| NLRP3 | 6 | Cluster 0 | 2.70 |
| VEGFA | 14 | Cluster 0 | 3.73 |
