# Supplementary material for: Humoral immunity and transcriptome differences of COVID-19 inactivated vacciane and protein subunit vaccine as third booster dose in human
Source: Front Immunol. 2022 Oct 21;13:1027180. doi: 10.3389/fimmu.2022.1027180 (PMC9634958; doi:10.3389/fimmu.2022.1027180)
Supplement: Supplementary file 8 [file Table_8.doc]

Table. S8 GO term of three cluster up-regulated gene sets.

| **Cluster** | **TermID** | **Name** | **Dispensability** | **GeneRatio** | **p.adjust** | **geneID** |
| --- | --- | --- | --- | --- | --- | --- |
| IV_group | GO:0001228 | DNA-binding transcription activator activity, RNA polymerase II-specific | 0.00 | 0.36 | 1.00E-03 | DLX2, PBX1, FOXC1, MEIS1 |
| IV_group | GO:0042379 | chemokine receptor binding | 0.68 | 0.18 | 3.22E-03 | CXCL5, PPBP |
| IV_group | GO:0030546 | signaling receptor activator activity | 0.00 | 0.27 | 8.99E-03 | IL10, CXCL5, PPBP |
| IV_group | GO:0070742 | C2H2 zinc finger domain binding | 0.03 | 0.09 | 2.44E-02 | EBF1 |
| IV_group | GO:0008301 | DNA binding, bending | 0.03 | 0.09 | 2.51E-02 | FOXC1 |
| IV_group | GO:0001664 | G protein-coupled receptor binding | 0.33 | 0.18 | 2.51E-02 | CXCL5, PPBP |
| IV_group | GO:0015144 | carbohydrate transmembrane transporter activity | 0.00 | 0.09 | 3.66E-02 | PPBP |
| IV_group | GO:0017046 | peptide hormone binding | 0.00 | 0.09 | 4.67E-02 | LEPR |
| IV_group | GO:1990841 | promoter-specific chromatin binding | 0.03 | 0.09 | 5.14E-02 | FOXC1 |
| IV_group | GO:0042562 | hormone binding | 0.03 | 0.09 | 7.03E-02 | LEPR |
| IV_group | GO:0003727 | single-stranded RNA binding | 0.15 | 0.09 | 7.03E-02 | DLX2 |
| IV_group | GO:0004896 | cytokine receptor activity | 0.18 | 0.09 | 7.39E-02 | LEPR |
| IV_group and PSV_group | GO:0001653 | peptide receptor activity | 0.00 | 0.14 | 6.75E-02 | GPR75, HCRTR1 |
| IV_group and PSV_group | GO:0015276 | ligand-gated ion channel activity | 0.00 | 0.14 | 6.75E-02 | KCNJ9, CHRNB2 |
| IV_group and PSV_group | GO:0008528 | G protein-coupled peptide receptor activity | 0.62 | 0.14 | 6.75E-02 | GPR75, HCRTR1 |
| IV_group and PSV_group | GO:0042166 | acetylcholine binding | 0.00 | 0.07 | 7.13E-02 | CHRNB2 |
| IV_group and_PSV_group | GO:0042165 | neurotransmitter binding | 0.02 | 0.07 | 7.13E-02 | CHRNB2 |
| IV_group and PSV_group | GO:1901338 | catecholamine binding | 0.03 | 0.07 | 7.13E-02 | ADRA2B |
| IV_group and PSV_group | GO:0042974 | retinoic acid receptor binding | 0.03 | 0.07 | 7.13E-02 | RARG |
| IV_group and PSV_group | GO:0015464 | acetylcholine receptor activity | 0.52 | 0.07 | 7.13E-02 | CHRNB2 |
| PSV_group | GO:0019956 | chemokine binding | 0.31 | 0.31 | 1.01E-09 | CCR2, CCR5, CXCR1, CX3CR1, CXCR2 |
| PSV_group | GO:0019955 | cytokine binding | 0.34 | 0.38 | 1.19E-08 | CCR2, CCR5, IFNGR1, CXCR1, CX3CR1, CXCR2 |
| PSV_group | GO:0140375 | immune receptor activity | 0.50 | 0.38 | 1.19E-08 | CCR2, CCR5, IFNGR1, CXCR1, CX3CR1, CXCR2 |
| PSV_group | GO:0001618 | virus receptor activity | 0.00 | 0.19 | 2.72E-04 | HSPA1A, CCR5, HSPA1B |
| PSV_group | GO:0140272 | exogenous protein binding | 0.03 | 0.19 | 2.72E-04 | HSPA1A, CCR5, HSPA1B |
| PSV_group | GO:0051787 | misfolded protein binding | 0.31 | 0.13 | 1.43E-03 | HSPA1A, HSPA1B |
| PSV_group | GO:0044183 | protein folding chaperone | 0.00 | 0.13 | 2.09E-03 | HSPA1A, HSPA1B |
| PSV_group | GO:0047485 | protein N-terminus binding | 0.33 | 0.13 | 1.46E-02 | HSPA1A, HSPA1B |
| PSV_group | GO:0042826 | histone deacetylase binding | 0.35 | 0.13 | 1.58E-02 | HSPA1A, HSPA1B |
| PSV_group | GO:0051082 | unfolded protein binding | 0.48 | 0.13 | 1.70E-02 | HSPA1A, HSPA1B |
| PSV_group | GO:0031072 | heat shock protein binding | 0.39 | 0.13 | 1.71E-02 | HSPA1A, HSPA1B |
| PSV_group | GO:0036312 | phosphatidylinositol 3-kinase regulatory subunit binding | 0.03 | 0.06 | 2.55E-02 | PIK3R1 |
| PSV_group | GO:0043560 | insulin receptor substrate binding | 0.27 | 0.06 | 2.55E-02 | PIK3R1 |
| PSV_group | GO:0005159 | insulin-like growth factor receptor binding | 0.28 | 0.06 | 3.69E-02 | PIK3R1 |
| PSV_group | GO:0019207 | kinase regulator activity | 0.00 | 0.13 | 3.96E-02 | CISH, PIK3R1 |
| PSV_group | GO:0019789 | SUMO transferase activity | 0.01 | 0.06 | 4.11E-02 | RANBP2 |
| PSV_group | GO:0005123 | death receptor binding | 0.48 | 0.06 | 4.41E-02 | FASLG |
| PSV_group | GO:0017046 | peptide hormone binding | 0.03 | 0.09 | 4.67E-02 | LEPR |
| PSV_group | GO:0004435 | phosphatidylinositol phospholipase C activity | 0.02 | 0.06 | 5.16E-02 | CCR5 |
| PSV_group | GO:0043548 | phosphatidylinositol 3-kinase binding | 0.30 | 0.06 | 5.72E-02 | PIK3R1 |
| PSV_group | GO:0097718 | disordered domain specific binding | 0.29 | 0.06 | 5.72E-02 | HSPA1A |
| PSV_group | GO:0030159 | signaling receptor complex adaptor activity | 0.00 | 0.06 | 6.82E-02 | PIK3R1 |
| PSV_group | GO:0001784 | phosphotyrosine residue binding | 0.32 | 0.06 | 6.82E-02 | PIK3R1 |
| PSV_group | GO:0015026 | coreceptor activity | 0.00 | 0.06 | 7.13E-02 | CCR5 |
