# Supplementary material for: Humoral immunity and transcriptome differences of COVID-19 inactivated vacciane and protein subunit vaccine as third booster dose in human
Source: Front Immunol. 2022 Oct 21;13:1027180. doi: 10.3389/fimmu.2022.1027180 (PMC9634958; doi:10.3389/fimmu.2022.1027180)
Supplement: Supplementary file 9 [file Table_9.doc]

Table. S9 GO term of three cluster down-regulated gene sets.

| **Cluster** | **TermID** | **Name** | **Dispensability** | **GeneRatio** | **p.adjust** | **geneID** |
| --- | --- | --- | --- | --- | --- | --- |
| IV_group | GO:0017070 | U6 snRNA binding | 0.32 | 0.09 | 5.54E-03 | LSM2, LSM3 |
| IV_group | GO:0017069 | snRNA binding | 0.00 | 0.09 | 2.98E-02 | LSM2, LSM3 |
| IV_group and PSV_group | GO:0030546 | signaling receptor activator activity | 0.00 | 0.58 | 1.53E-07 | CCL20, IL1A, IL1B, CXCL8, CXCL2, TNF, EGF |
| IV_group and PSV_group | GO:0005149 | interleukin-1 receptor binding | 0.59 | 0.17 | 5.52E-04 | IL1A, IL1B |
| IV_group and PSV_group | GO:0045236 | CXCR chemokine receptor binding | 0.68 | 0.17 | 5.52E-04 | CXCL8, CXCL2 |
| IV_group and PSV_group | GO:0070851 | growth factor receptor binding | 0.36 | 0.25 | 7.65E-04 | IL1A, IL1B, EGF |
| IV_group and PSV_group | GO:0002020 | protease binding | 0.00 | 0.17 | 2.15E-02 | TNFAIP3, TNF |
| PSV_group | GO:0019955 | cytokine binding | 0.32 | 0.30 | 2.96E-04 | CCRL2, CXCR4, IL1RN |
| PSV_group | GO:0019956 | chemokine binding | 0.33 | 0.20 | 5.25E-04 | CCRL2, CXCR4 |
| PSV_group | GO:0045236 | CXCR chemokine receptor binding | 0.50 | 0.17 | 5.52E-04 | CXCL8, CXCL2 |
| PSV_group | GO:0070851 | growth factor receptor binding | 0.58 | 0.25 | 7.65E-04 | IL1A, IL1B, EGF |
| PSV_group | GO:0004896 | cytokine receptor activity | 0.55 | 0.20 | 3.64E-03 | CCRL2, CXCR4 |
| PSV_group | GO:0140375 | immune receptor activity | 0.50 | 0.20 | 6.65E-03 | CCRL2, CXCR4 |
| PSV_group | GO:0001653 | peptide receptor activity | 0.58 | 0.20 | 6.96E-03 | CCRL2, CXCR4 |
| PSV_group | GO:0008528 | G protein-coupled peptide receptor activity | 0.62 | 0.20 | 6.96E-03 | CCRL2, CXCR4 |
| PSV_group | GO:0005539 | glycosaminoglycan binding | 0.00 | 0.20 | 1.39E-02 | NLRP3, VEGFA |
| PSV_group | GO:0005172 | vascular endothelial growth factor receptor binding | 0.72 | 0.10 | 1.56E-02 | VEGFA |
| PSV_group | GO:0005161 | platelet-derived growth factor receptor binding | 0.28 | 0.10 | 1.59E-02 | VEGFA |
| PSV_group | GO:0042834 | peptidoglycan binding | 0.21 | 0.10 | 1.76E-02 | NLRP3 |
| PSV_group | GO:0001968 | fibronectin binding | 0.03 | 0.10 | 2.63E-02 | VEGFA |
| PSV_group | GO:0042056 | chemoattractant activity | 0.59 | 0.10 | 3.21E-02 | VEGFA |
| PSV_group | GO:0030547 | signaling receptor inhibitor activity | 0.00 | 0.10 | 3.60E-02 | IL1RN |
| PSV_group | GO:0015026 | coreceptor activity | 0.00 | 0.10 | 3.76E-02 | CXCR4 |
