# Supplementary material for: Humoral immunity and transcriptome differences of COVID-19 inactivated vacciane and protein subunit vaccine as third booster dose in human
Source: Front Immunol. 2022 Oct 21;13:1027180. doi: 10.3389/fimmu.2022.1027180 (PMC9634958; doi:10.3389/fimmu.2022.1027180)
Supplement: Supplementary file 10 [file Table_10.doc]

Table. S10 The KEGG term of gene sets in greenyellow module.

| **ID** | **Term** | **Term PValue** | **Associated Genes Found** |
| --- | --- | --- | --- |
| KEGG:04657 | IL-17 signaling pathway | 6.41E-08 | CCL20, CXCL2, CXCL3, CXCL8, IL17RB, IL1B, PTGS2, TNF, TNFAIP3 |
| KEGG:04668 | TNF signaling pathway | 2.95E-07 | CCL20, CSF1, CXCL2, CXCL3, IL1B, IRF1, PTGS2, TNF, TNFAIP3 |
| KEGG:05323 | Rheumatoid arthritis | 8.39E-07 | CCL20, CSF1, CXCL2, CXCL3, CXCL8, HLA-DMA, IL1B, TNF |
| KEGG:04064 | NF-kappa B signaling pathway | 1.98E-06 | BCL10, CXCL2, CXCL3, CXCL8, IL1B, PTGS2, TNF, TNFAIP3 |
| KEGG:05133 | Pertussis | 2.70E-06 | C1QA, CXCL8, GNAI3, IL1B, IRF1, NLRP3, TNF |
| KEGG:05134 | Legionellosis | 6.68E-06 | CXCL2, CXCL3, CXCL8, IL1B, RAB1A, TNF |
| KEGG:04061 | Viral protein interaction with cytokine and cytokine receptor | 1.69E-05 | CCL20, CSF1, CXCL2, CXCL3, CXCL8, IL37, TNF |
| KEGG:04625 | C-type lectin receptor signaling pathway | 2.18E-05 | BCL10, IL1B, IRF1, NLRP3, PLK3, PTGS2, TNF |
| KEGG:05142 | Chagas disease | 1.85E-04 | C1QA, CXCL8, GNAI3, IL1B, PPP2CA, TNF |
| KEGG:05146 | Amoebiasis | 1.50E-03 | CXCL2, CXCL3, CXCL8, IL1B, TNF |
| KEGG:05140 | Leishmaniasis | 3.69E-03 | HLA-DMA, IL1B, PTGS2, TNF |
| KEGG:05332 | Graft-versus-host disease | 4.94E-03 | HLA-DMA, IL1B, TNF |
| KEGG:04940 | Type I diabetes mellitus | 5.28E-03 | HLA-DMA, IL1B, TNF |
| KEGG:05144 | Malaria | 8.05E-03 | CXCL8, IL1B, TNF |
| KEGG:04640 | Hematopoietic cell lineage | 8.96E-03 | CSF1, HLA-DMA, IL1B, TNF |
| KEGG:04933 | AGE-RAGE signaling pathway in diabetic complications | 9.28E-03 | CXCL8, F3, IL1B, TNF |
| KEGG:05321 | Inflammatory bowel disease | 1.64E-02 | HLA-DMA, IL1B, TNF |
| KEGG:05120 | Epithelial cell signaling in Helicobacter pylori infection | 2.00E-02 | CXCL2, CXCL3, CXCL8 |
